# Managing Challenging Behaviour in a Patient With Learning Disability and Autism Spectrum Disorder With Trazodone: A Clinical Case

**DOI:** 10.1192/bjo.2026.11876

**Published:** 2026-06-30

**Authors:** Gbenga Popoola, Kehinde Omotayo

**Affiliations:** Mersey Care NHS Foundation Trust, Liverpool, United Kingdom

## Abstract

**Aims:**

Challenging behaviours in individuals with intellectual disability and autism spectrum disorder are complex, multifactorial phenomena that often result in significant distress for patients, families, and caregivers. They are associated with poor quality of life, increased use of mental health services, and heightened risk of trauma for carers. Pharmacological management has traditionally favoured antipsychotics, with limited evidence supporting the use of antidepressants.

**Methods:**

We report the case of a 26-year-old woman with moderate learning disability and autism spectrum disorder who exhibited severe challenging behaviours, including self-harm, aggression and property damage. These behaviours persisted despite trials of benzodiazepines, analgesia, and multidisciplinary behavioural interventions. Antipsychotics were avoided due to comorbid pituitary tumour.

Following a clinical review that identified anxiety as a perpetuating factor, trazodone was introduced and titrated to 50 mg three times daily.

**Results:**

Initiation of trazodone has resulted in marked reduction in the frequency and severity of challenging behaviours, improved identification of triggers, and enhanced engagement in structured ward activities. Residual incidents are shorter, less severe, and easily redirected, with fewer requiring physical intervention. The patient demonstrated improved participation in therapeutic activities, and staff reported greater confidence in managing residual behaviours.

**Conclusion:**

This case highlights the potential role of trazodone, an antidepressant, in managing challenging behaviours in individuals with intellectual disability and autism spectrum disorder. It underscores the importance of individualized treatment approaches and contributes to the limited evidence base supporting alternative psychotropic strategies beyond antipsychotics. Further studies are needed to assess antidepressant efficacy in managing challenging behaviours.

